# U-Curve Association between Timing of Renal Replacement Therapy Initiation and In-Hospital Mortality in Postoperative Acute Kidney Injury

**DOI:** 10.1371/journal.pone.0042952

**Published:** 2012-08-28

**Authors:** Chih-Chung Shiao, Wen-Je Ko, Vin-Cent Wu, Tao-Min Huang, Chun-Fu Lai, Yu-Feng Lin, Chia-Ter Chao, Tzong-Shinn Chu, Hung-Bin Tsai, Pei-Chen Wu, Guang-Huar Young, Tze-Wah Kao, Jenq-Wen Huang, Yung-Ming Chen, Shuei-Liong Lin, Ming-Shou Wu, Pi-Ru Tsai, Kwan-Dun Wu, Ming-Jiuh Wang

**Affiliations:** 1 Division of Nephrology, Department of Internal Medicine, Saint Mary’s Hospital Luodong, and Saint Mary’s Medicine, Nursing and Management College, Yilan, Taiwan; 2 Department of Traumatology, National Taiwan University Hospital, Taipei, Taiwan; 3 Department of Surgery, National Taiwan University Hospital, Taipei, Taiwan; 4 Division of Nephrology, Department of Internal Medicine, National Taiwan University Hospital, Taipei, Taiwan; 5 Division of Nephrology, Department of Internal Medicine, National Taiwan University Hospital Yun-Lin Branch, Douliu City, Yunlin County, Taiwan; 6 Department of Anesthesiology and Forensic Medicine, National Taiwan University Hospital, Taipei, Taiwan; University of Sao Paulo Medical School, Brazil

## Abstract

**Background:**

Postoperative acute kidney injury (AKI) is associated with poor outcomes in surgical patients. This study aims to evaluate whether the timing of renal replacement therapy (RRT) initiation affects the in-hospital mortality of patients with postoperative AKI.

**Methodology:**

This multicenter retrospective observational study, which was conducted in the intensive care units (ICUs) in a tertiary hospital (National Taiwan University Hospital) and its branch hospitals in Taiwan between January, 2002, and April, 2009, included adult patients with postoperative AKI who underwent RRT for predefined indications. The demographic data, comorbid diseases, types of surgery and RRT, and the indications for RRT were documented. Patients were categorized according to the period of time between the ICU admission and RRT initiation as the early (EG, ≦1 day), intermediate (IG, 2–3 days), and late (LG, ≧4 days) groups. The in-hospital mortality rate censored at 180 day was defined as the endpoint.

**Results:**

Six hundred forty-eight patients (418 men, mean age 63.0±15.9 years) were enrolled, and 379 patients (58.5%) died during the hospitalization. Both the estimated probability of death and the in-hospital mortality rates of the three groups represented U-curves. According to the Cox proportional hazard method, LG (hazard ratio, 1.527; 95% confidence interval, 1.152–2.024; *P = *0.003, compared with IG group), age (1.014; 1.006–1.021), diabetes (1.279; 1.022–1.601; *P = *0.031), cirrhosis (2.147; 1.421–3.242), extracorporeal membrane oxygenation support (1.811; 1.391–2.359), initial neurological dysfunction (1.448; 1.107–1.894; *P* = 0.007), pre-RRT mean arterial pressure (0.988; 0.981–0.995), inotropic equivalent (1.006; 1.001–1.012; *P* = 0.013), APACHE II scores (1.055; 1.037–1.073), and sepsis (1.939; 1.536–2.449) were independent predictors of the in-hospital mortality (All *P*<0.001 except otherwise stated).

**Conclusions:**

The current study found a U-curve association between the timing of the RRT initiation after the ICU admission and patients’ in-hospital mortalities, and alerts physicians of certain factors affecting the outcome after the RRT initiation.

## Introduction

Postoperative acute kidney injury (AKI), with rates of occurrence ranging from 0.8 to 30% due to the various definitions of the pathology and the different associated surgery types, is associated with not only the increased healthcare costs and duration of hospital stay, but also higher morbidity and mortality rates [1], [2], [3]. Among the critically ill patients admitted to the intensive care unit (ICU), approximately 4–15% develop AKI that requires renal replacement therapy (RRT), and AKI is associated with a significant in-hospital mortality rate of 50–80% [4], [5]. To date, RRT remains a critical supportive measure for AKI [6]. The goal of RRT is to maintain solute clearance and fluid balance to prevent subsequent multi-organ damage as renal function recovers. However, there are limited data documenting the significant factors used to determine if and when it is appropriate to initiate RTT in this setting. Because of the great impact of RRT-requiring AKI on patients’ prognoses, many investigators have made every effort to define the optimal timing of RRT initiation.

Although some meta-analyses have revealed the probable beneficial effects of “earlier” initiation of RRT for AKI [7], [8], the non-standardized triggers for this therapy and the heterogeneous populations represented in the published studies preclude the possibility of forming a definitive recommendation. Besides the traditional markers, other parameters, including the duration of time between admission to the ICU and the initiation of RRT [9], were used to determine the timing of the RRT. A prospective, observational study by Bagshaw *et al.* found that RRT initiation within 2 days of the ICU admission was associated with better survival compared to the patients who received RRT after 5 days in the unit [9]. The beneficial effect of earlier RRT was also seen in other studies, but their cutoff points relative to the ICU admission varied (*e.g*., “on admission” versus “at 24 hours” by Andrade *et al.* [10], “<2 days” versus “≧2 days” by Payen *et al.* [11], and “<3 days” versus “≧3 days” by Ostermann *et al.* [12]). Furthermore, the association between the temporal relationship and the outcomes was not supported by a prospective, randomized trial, in which no survival difference was observed between patients starting RRT at a mean time of 1.5 days and 3 days after the ICU admission [13]. The heterogeneity of the surgical/medical patient population included in these investigations may also explain the discordant results generated, as different pathologies have varying influences on the prognoses for AKI [14], [15]. Additionally, the different patient categories based on the time to the initiation of the RRT in these studies may have also played a role in the lack of a general consensus in the results. Regardless, simply categorizing all the participants into 2 or 3 groups accordingly may not truly reflect the complete clinical scenarios.

Thus, we designed this study to evaluate the association between the timing of RRT relative to the ICU admission and the patients’ in-hospital mortality rate. We also analyzed the factors that may predict the mortality in the setting of postoperative AKI.

## Methods

### Study Design and Participants

This multicenter, retrospective observational study was conducted using the NSARF (National Taiwan University Hospital Study Group on Acute Renal Failure) database in which the data was prospectively collected in the ICUs in a tertiary hospital (National Taiwan University Hospital) and its branch hospitals in Taiwan for quality and outcome assurance [16], [17], [18]. The Institutional Review Board of National Taiwan University Hospital approved the study (No. 31 MD03) and determined that there was no need for informed consent as there was neither breach of privacy nor interference with clinical decisions. The data were analyzed anonymously as well.

Adult patients who underwent major surgeries with postoperative AKI requiring RRT in the ICUs between January 1, 2002, and April 30, 2009, were eligible for this study. The exclusion criteria included undergoing chronic dialysis, less than 18 years of age, receiving RRT before the surgery. The enrolled patients were categorized into three groups based on the duration of time between the ICU admission and the initiation of the RRT. The patients were designated as follows: the early group (EG, defined as 0–1 day), the intermediate group (IG, 2–3 days), and the late group (LG, ≧4 days). Under the same practice principles, those enrolled in this study were treated and followed until November 30, 2009.

The baseline demographic data, comorbid diseases, types of surgery and RRT, and indications for RRT were documented. Biochemical data, such as the complete blood cell count, the blood urea nitrogen (BUN), the serum creatinine (sCr), the estimated glomerular filtration rate (eGFR) calculated using the Chinese Modification of Diet in Renal Disease equation [19], the serum albumin, and the serum potassium, were recorded when the patient was admitted to the ICU and when RRT was initiated. The severity scores, including the Glasgow Coma Scale (GCS) scores, the Acute Physiology and Chronic Health Evaluation II (APACHE II) scores [20], and the Sequential Organ Failure Assessment (SOFA) scores [21], were also measured at the two time points. Mechanical ventilation (MV), extracorporeal membrane oxygenation (ECMO) support, inotropic equivalents [IE,  =  dopamine + dobutamine + (epinephrine + norepinephrine + isoproterenol) × 100+ milrinone ×15 (mcg/kg/min)] [22], and net fluid balance (% body weight [BW],  =  [pre-RRT BW − baseline BW]/baseline BW) at RRT initiation were recorded as well.

The baseline sCr was determined as the last value measured one month to one year prior to the index admission [23], or the lowest sCr value before operation during the index admission [24]. The peak sCr was defined as the highest sCr before the initiation of RRT in the ICUs. The predefined indications for RRT initiation at our institutes were as follows: (1) azotemia (BUN>80 mg/dl and sCr>2 mg/dl) with uremic symptoms (encephalopathy, pericarditis, pleuritis); (2) oliguria (urine amount <100 mL/8 h) or anuria refractory to diuretics; (3) fluid overload refractory to diuretics use with a central venous pressure (CVP)>12 mmHg; (4) hyperkalemia (serum potassium>5.5 mmol/L) refractory to medical treatment; and (5) metabolic acidosis (a pH <7.2 in arterial blood gas) [17], [25]. Other definitions were made as followings: diabetes mellistus (DM), previous usage of insulin or oral hypoglycemic agents; hypertension, usage of anti-hypertension agents or blood pressure>145/95 mmHg at the time of hospitalization; heart failure, New York Heart Association functional class IV; chronic kidney disease (CKD), baseline eGFR ≦45 ml/min/1.73 m^2^ for more than 3 months [26]; neurological dysfunction, GCS <9 points [27]; sepsis, persisted or progressive signs and symptoms of the systemic inflammatory response syndrome with clinical evidence of infection [28]; RRT wean-off, cessation from RRT for at least 30 days [18]. Besides, major surgeries were defined if the length of hospital stay for patients in a given diagnosis-related group exceeded 2 days [18], [29], and which were divided into 5 categories, namely, neurosurgery, cardiovascular surgery (CVS), chest surgery (defined as surgery in the chest area excluding CVS and thoracic vascular surgery), abdominal surgery, and others.

The modalities of the RRT for individual patients were initially chosen and may have been subsequently changed depending on the patients’ hemodynamic stability. For those who needed an IE of more than 15 mcg/kg/min to maintain a systemic blood pressure up to 120 mmHg, continuous venovenous hemofiltration (CVVH) was performed using the high-flux filters (Hemofilter, PAN-10, Asahi Kasei, Japan) and the HF-400 (Informed, Geneva, Switzerland). The hemofiltration and blood flows were 35 ml/kg/hr and 200 ml/min, respectively. The replacement fluid was bicarbonate-buffered and was predilutionally administered at a dynamically adjusted rate to achieve the desired fluid therapy goals. The default fluid was composed of Na 142 mEq/L, bicarbonate 33 mEq/L, Ca 2.6 mEq/L, and Mg 1.4 mEq/L. For patients with a required IE of 5–15 mcg/kg/min, sustained low efficiency daily dialysis (SLEDD) or diafiltration (SLEDD-f) was used with a blood flow of 200 ml/min, a dialysate flow of 300 ml/min, and a hemofiltration flow of 35 ml/kg/hour. The duration of the filtration procedures was approximately 6–12 hrs, depending on the amount of ultrafiltration. Intermittent hemodialysis was performed for 4 hours except for the first and second sessions using the low-flux polysulfone hemofilters (KF-18C, Kawasumi Laboratories, Japan) with dialysate and blood flows of 500 ml/min and 200 ml/min, respectively. [16], [18], [25]. Double lumen catheters were placed for vascular accesses.

### Study Outcomes

The endpoint of this study was in-hospital mortality censored at 180 days. The censoring period was calculated from the RRT initiation to the mortality (in non-survivors) or the hospital discharge (in survivors).

### Statistical Methods

The statistical analyses were performed using the *R* 2.12.1 (R Foundation for Statistical Computing, Vienna, Austria) software. The continuous data were expressed as mean ± standard deviation unless otherwise specified and compared using the Kruskal-Wallis Rank Sum Test or Wilcoxon Rank Sum Test with Bonferroni correction. The categorical variables were shown as numbers (percentages) and analyzed using Fisher’s Exact Test with Bonferroni correction. The Kaplan-Meier survival curves with the log-rank test were drawn to show the differences in patient survival among the three groups. The generalized additive models (GAM) were applied to measure the probability of death according to the individual calendar day from the ICU admission to the RRT initiation. We then analyzed the independent predictors of in-hospital mortality with the stepwise selection method of the Cox proportional hazards model, and identified predictors for the EG and LG groups by the logistic regression method. All the variables were selected for multivariate analysis if they had a *P*≦0.1 on univariate analysis or if they are considered to be clinically important. The basic model-fitting techniques for variable selection, goodness-of-fit assessment, and regression diagnostics were used in our regression analyses to ensure the quality of analysis results. Specifically, we used the stepwise variable selection procedure with both significance level for entry and significance level for stay set to 0.15 or greater to select the relevant covariates for the final Cox proportional hazards model. In all statistical analyses, a two-sided *p*≦0.05 was considered statistically significant.

## Results

**Figure 1:**
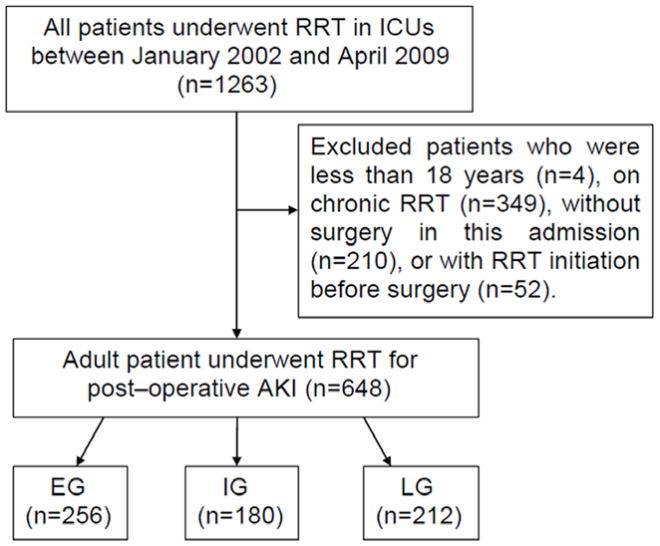
Approach to selecting and categorizing patients. EG, IG, and LG were defined as ≦ 1 day, 2–3 days, and ≧ 4 days between ICU admission and RRT initiation, respectively. Abbreviations: AKI, acute kidney injury; EG, early group; ICU, intensive care unit; IG, intermediate group; LG, late group; RRT, renal replacement therapy.

Figure 1 shows the flowchart of patient selecting and categorizing. After screening of 1263 patients, 648 (418 men, mean age 63.0±15.9 years) were enrolled and categorized into EG (n = 256), IG (n = 180), and LG (n = 212). There were 20 patients (3.1%) undergoing neurosurgery, 58 (8.9%) with chest surgery, 347 (53.5%) with CVS, 183 (28.2%) with abdominal surgery, and 40 (6.2%) with other surgeries. The indications for RRT included 358 patients (55.2%) for azotemia with uremic symptoms, 408 (63.0%) for fluid overload, 543 (83.8%) for oliguria, and 80 (12.3%) for hyperkalemia or acidosis. Most of our patients had more than one indication for RRT initiation.

**Figure 2:**
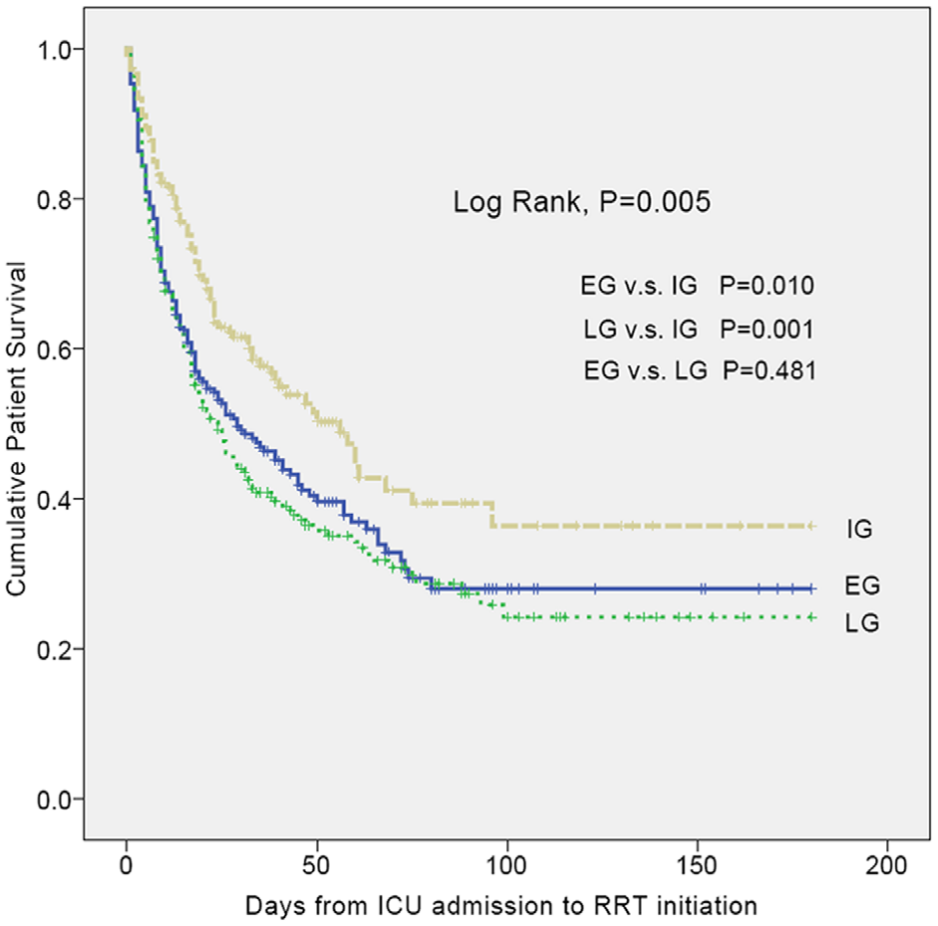
Estimated survival probability among EG, IG, and LG. The survival period was calculated from RRT initiation. EG (blue line, n = 243), IG (green line, n = 146), and LG (red line, n = 227) were defined as RRT initiation ≦ 1 days, 2–3 days, and ≧ 4 days after ICU admission, respectively. Abbreviations: EG, early group; ICU, intensive care unit; IG, intermediate group; LG, late group; RRT, renal replacement therapy.

**Figure 3:**
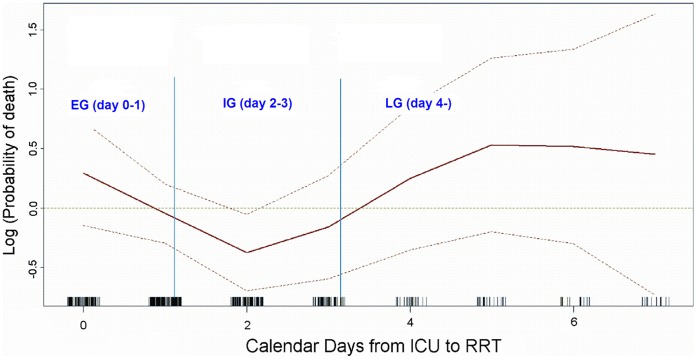
Probability of death by the calendar days from ICU admission to RRT initiation. The figure was drawn using generalized additive model. Adjusted by sex, age, diabetes mellitus, chronic kidney disease, cirrhosis, extracorporeal membrane oxygenation support, initial neurological dysfunction, as well as sepsis, mean arterial pressure, inotropic equivalent, and Acute Physiology and Chronic Health Evaluation II scores at RRT initiation. Abbreviations: EG, early group; ICU, intensive care unit; IG, intermediate group; LG, late group; RRT, renal replacement therapy.

A total of 379 patients (58.5%) died during hospitalization period within 180 days. By Kaplan-Meier method, we demonstrated the much lower survival proportion of both EG and LG groups as compared with IG (Log Rank, *P = *0.005) (Figure 2). While both the estimated probability of death estimated using GAM (Figure 3) and in-hospital mortality rates of the 3 groups (59.0%, 47.8%, and 67.0%, consecutively. figure not shown) represented U-shaped curves.

### Parameters between EG, IG, and LG (Tables 1 and S1)

The patients in the EG had higher proportion of CKD (*P = *0.015), CVS, and ECMO support, but less chest and abdominal surgery (*P = *0.001). They had the worst conditions at ICU admission among the 3 groups, including the severity scores (highest APACHE II and SOFA scores), neurological function (lowest GCS), hemodynamics [highest IE and CVP level, with lowest mean arterial pressure (MAP)], and renal function (highest BUN and sCr, and lowest eGFR). Subsequently, they received RRT soonest, with a mean period of only 0.6 days after ICU admission, for the indications of oliguria/anuria rather than azotemia.

**Table 1:** Comparisons of demographic data and clinical parameters among the three groups.

Variable	EG ∣ (*n* = 256)	IG (*n* = 180)	LG∥ (*n* = 212)	P-value
**Demographic data**				
Age, years	61.3±14.7	62.2±16.5	65.7±16.5*	0.009
Man	169 (66.0)	114 (63.3)	135 (63.7)	0.808
DM	90 (35.2)	60 (33.3)	68 (32.1)	0.777
CKD	139 (54.3)**	78 (43.3)	90 (42.5)	0.015
Heart failure	12 (4.7)	11 (6.1)	13 (6.1)	0.738
Cirrhosis	9 (3.5)**	19 (10.6)	13 (6.1)	0.012
Initial neurological dysfunction	55 (21.5)	27 (15.0)	44 (20.8)	0.204
Sepsis at RRT	43 (16.8)	31 (17.2)	68 (32.1)**	<0.001
Sepsis at death¥	81 (53.6)	52 (61.2)	110 (77.5)**	<0.001
ECMO support	92 (35.9)**	32 (17.8)	43 (20.3)	<0.001
Mechanical Ventilation	240 (93.8)	163 (90.6)	204 (96.2)	0.071
CVVH as initial mode	197 (77.0)**	100 (55.6)	113 (53.3)	<0.001
Hospital stay, days	43.0±43.6*	52.5±53.7	62.8±50.9	<0.001
ICU to RRT, days	0.6±0.5**	2.6±0.7	17.9±24.5**	<0.001
RRT to death/discharge, days	31.2±33.9	38.8±43.5	34.6±36.5	0.111
**Surgery category**				<0.001
Neurosurgery	4 (1.6)	7 (3.9)	9 (4.2)	0.189
Chest surgery	5 (2.0)*	12 (6.7)	$1 (19.3)**	<0.001
Cardiovascular surgery	185 (72.3)**	86 (47.8)	76 (35.8)*	<0.001
Abdominal surgepy	51 (19.9)**	63 (35.0)	69 (32.5)	0.001
Others	11 (4.3)	12 (6.7)	17 (8.0)	0.237
**Data at RRT initiation**				
RIFLE-I & -F	69 (27.0)**	119 (66.1)	142 (67.0)	<0.001
MAP, mmHg	76.5±16.8**	84.5±16.1	82.0±15.3	<0.001
BUN, mg/dL	49.0±28.7**	58.6±25.9	90.1±43.3**	<0.001
Creatinine, mg/dL	3.0±1.8**	3.8±1.9	3.3±1.7*	<0.001
eGFR, ml/min/1.73 m^2^	29.7±19.3**	21.7±15.7	26.6±22.8*	<0.001
CVP, mmHg	13.4±5.6	14.5±5.4	13.7±5.3	0.136
IE, mcg/kg/min	19.9±22.1*	14.9±19.7	9.6±12.5**	<0.001
GCS scores	11.3±4.8	12.2±4.3	11.1±4.4	0.169
APACHE II scores	12.9±6.6	12.2±6.2	12.8±5.7	0.535
SOFA scores	11.4±3.6	11.5±3.6	11.3±3.9	0.856
**Indications for RRT**				
Azotemia with uremic symptoms^a^	115 (44.9)	98 (54.4)	145 (68.4)**	<0.001
Fluid overload^b^	163 (63.7)	122 (67.8)	123 (58.0)	0.131
Oliguria or anuria^c^	237 (92.6)	162 (90.0)	144 (67.9)**	<0.001
Hyperkalemia or acidosis^d^	30 (11.7)	16 (8.9)	34 (16.1)	0.089
**In-hospital mortality**	151 (59.0)*	85 (47.8)	14 (67.0)**	0.001

**Notes:** To save the space, data with less clinical relevance, without statistical significance, and at ICU admission were presented in the online supporting tables only.

EG, ≦ 1 day; IG, 2–3 days; LG, ≧ 4 days between ICU admission and RRT initiation. Values are presented as mean ± standard deviation or number (percentage) unless otherwise stated. P-value was calculated using Kruskal-Wallis Rank Sum Test, Wilcoxon Rank Sum Tests in two sample comparison with Bonferroni correction and Fisher’s Exact Test for count data.

¥ the percentage was calculated dividing by number of the deaths;

**∣** EG versus IG;

**∥** LG versus IG;

* P<0.05;

** P<0.01.

a azotemia was defined as BUN>80 mg/dl and creatinine>2 mg/dl;

b fluid overload means CVP>12 mmHg;

c oliguria was defined as urine output <100 ml/8 hr with diuretics use;

d hyperkalemia denotes serum potassium>5.5 mmol/l, acidosis denotes pH<7.2 in arterial blood. RRT wean-off, cessation from RRT for at least 30 days.

**Abbreviations:** APACHE II, Acute Physiology and Chronic Health Evaluation II; BMI, body mass index; BUN, blood urea nitrogen; CKD, chronic kidney disease; CVP, central venous pressure; CVVH, continuous venous-venous hemofiltration; DM, diabetes mellitus; ECMO, extracorporeal membrane oxygenation; EG, early group; eGFR, estimated glomerular filtration rate; GCS, Glasgow Coma Scale; ICU, intensive care unit; IE, inotropic equivalent; IG, intermediate group; LG, late group; MAP, mean arterial pressure; RRT, renal replacement therapy; SOFA, Sequential Organ Failure Assessment; WBC, white blood cell.

On the contrary, the patients in the remaining two groups had lower severity of illness (lower APACHE II and SOFA), hemodynamics (lower CVP, IE, and higher MAP), and renal function (lower BUN, sCr, and higher eGFR) when they were admitted to ICU. However, their renal functions gradually got worse during the ICU admission, which made them start RRT after 2.6 days and 17.9 days in IG and LG, respectively, of ICU admission. At RRT initiation, although their hemodynamics were still better (lower IE and higher MAP), but renal function turned to be worse (higher BUN and sCr, lower eGFR, and higher proportion of RIFLE-I & F) than EG, while the severity scores and consciousness were not of statistical differences.

As to the patients in LG, they were older but had the best condition at ICU admission (lowest SOFA scores and IE, along with highest MAP and eGFR). They had higher percentage of receiving chest surgery and more sepsis at RRT which were initiated due to azotemia rather than oliguria/anuria, consequently accompanying a higher sepsis-associated mortality rate. (All *P*<0.001 except otherwise stated).

### Parameters between Survivors and Non-survivors (Tables 2 and S2)

In the comparisons between survivors and non-survivors, the non-survivors were older (*P = *0.014), along with higher proportion of initial neurological dysfunction, sepsis at RRT initiation, ECMO support, CVVH, MV (*P = *0.032), but less CKD. They underwent more chest surgery (*P = *0.026) but less neurosurgery (*P = *0.038), and had higher proportion of fluid overload (*P = *0.013) as indication for RRT than survivors.

**Table 2:** Comparisons of demographic data and clinical parameters between survivors and non-survivors.

Variable	Survivors (n = 269)	Non-survivors (n = 379)	P-value
**Demographic data**			
Age, years	61.2±15.8	64.3±15.8	0.014
Man	180(66.9)	238 (62.8)	0.317
DM	86 (39.4)	132 (34.8)	0.500
CKD	152 (56.5)	155 (40.9)	<0.001
Heart failure	19 (7.1)	17 (4.5)	0.167
Cirrhosis	14 (5.2)	27 (7.1)	0.413
Initial neurological dysfunction	29 (10.8)	97 (25.6)	<0.001
Sepsis at RRT	27 (10.0)	115 (30.3)	<0.001
ECMO support	49 (18.2)	118 (31.1)	<0.001
Mechanical Ventilation	245 (91.1)	362 (95.5)	0.032
CVVH as initial mode	142 (52.8)	268 (70.7)	<0.001
Hospital stay, days	74.2±57.1	36.1±35.7	<0.001
RRT to death/discharge, days	56.9±45.1	18.3±19.2	<0.001
**Surgery category**			0.051
Neurosurgery	13 (4.8)	7 (1.8)	0.038
Chest surgery	16 (5.9)	42 (11.1)	0.026
Cardiovascular surgery	145 (53.9)	202 (53.3)	0.936
Abdominal surgery	79 (29.4)	104 (27.4)	0.596
Others	16 (5.9)	24 (6.3)	0.870
**Data at RRT initiation**			
RIFLE-I & -F	143 (53.2)	187 (49.3)	0.340
MAP, mmHg	85.4±15.7	77.0±16.2	<0.001
Creatinine, mg/dL	3.8±2.0	3.0±1.6	<0.001
eGFR, ml/min/1.73 m^2^	22.6±16.3	29.2±21.7	<0.001
IE, mcg/kg/min	11.3±16.5	17.9±20.5	<0.001
GCS scores	13.1±3.4	10.2±4.9	<0.001
APACHE II scores	10.5±5.2	14.2±6.4	<0.001
SOFA scores	10.0±3.1	12.4±3.8	<0.001
**Indications for RRT**			
Azotemia with uremic symptoms^a^	153 (56.9)	205 (54.1)	0.521
Fluid overload^b^	154 (57.2)	254 (67.0)	0.013
Oliguria or anuria^c^	227 (84.4)	316 (83.4)	0.676
Hyperkalemia or acidosis^d^	25 (9.3)	55 (14.6)	0.052

**Notes:** To save the space, data with less clinical relevance, without statistical significance, and at ICU admission were presented in the online supporting tables only.

Values are presented as mean ± standard deviation or number (percentage) unless otherwise stated. P-value was calculated using Wilcoxon Rank Sum Tests for continuous data and Fisher’s Exact Test for count data.

a azotemia was defined as BUN>80 mg/dl and creatinine>2 mg/dl;

b fluid overload means CVP>12mmHg;

c oliguria was defined as urine output <100 ml/8 hr with diuretics use;

d hyperkalemia denotes serum potassium>5.5 mmol/l, acidosis denotes pH<7.2 in arterial blood. RRT wean-off, cessation from RRT for at least 30 days.

**Abbreviations:** APACHE II, Acute Physiology and Chronic Health Evaluation II; BMI, body mass index; BUN, blood urea nitrogen; CKD, chronic kidney disease; CVP, central venous pressure; CVVH, continuous venous-venous hemofiltration; DM, diabetes mellitus; ECMO, extracorporeal membrane oxygenation; EG, early group; eGFR, estimated glomerular filtration rate; GCS, Glasgow Coma Scale; ICU, intensive care unit; IE, inotropic equivalent; IG, intermediate group; LG, late group; MAP, mean arterial pressure; RRT, renal replacement therapy; SOFA, Sequential Organ Failure Assessment; WBC, white blood cell.

At ICU admission, they had worse hemodynamics [higher IE (*P = *0.013) with lower MAP (*P = *0.006)] and neurological function (GCS scores), and higher severity of illness [higher APACHE-II and SOFA scores (*P = *0.004)], but better renal function [lower sCr with higher eGFR]. The tendency persisted up to RRT initiation. (All *P*<0.001 except otherwise stated).

### Independent Predictors for In-hospital Mortality (Table 3)

Multivariate analysis was performed to determine the predictors for in-hospital mortality. The variables put into analysis included age, DM, liver cirrhosis, heart failure, baseline CKD, ECMO, MV, initial neurological dysfunction, sepsis and some parameters (RIFLE-I & F, MAP, IE, APACHE II scores) at RRT initiation, EG and LG as compared with IG, indications for RRT (azotemia, fluid overload & oliguria, and hyperkalemia & acidosis), as well as surgery types (neuro-, chest, cardiovascular, abdominal, and other surgery).

**Table 3:** Multivariate analysis of the predictors for in-hospital mortality by fitting multiple Cox proportional hazards model with the stepwise variable selection method.

Covariate	Regression Coefficient	Standard Error	*P*-Value	Hazard Ratio	95% CI
Late group^a^	0.423	0.144	0.003	1.527	1.152–2.024
Age^b^	0.014	0.004	<0.001	1.014	1.006–1.021
DM^c^	0.246	0.114	0.031	1.279	1.022–1.601
Cirrhosis^d^	0.764	0.210	<0.001	2.147	1.421–3.242
ECMO support^e^	0.594	0.135	<0.001	1.811	1.391–2.359
Initial neurological dysfunction^f^	0.370	0.137	0.007	1.448	1.107–1.894
Sepsisg,h	0.662	0.119	<0.001	1.939	1.536–2.449
MAPb,h	−0.012	0.004	0.001	0.988	0.981–0.995
IEb,h	0.006	0.003	0.013	1.006	1.001–1.012
APACHE II scoresb,h	0.053	0.009	<0.001	1.055	1.037–1.073

**Notes:** Variables put into multivariate analysis were selected if they had a *P*≦0.1 on univariate analysis or if they are thought to be important. They included age, DM, cirrhosis, heart failure, CKD, ECMO, mechanical ventilation, initial neurological dysfunction, sepsis and some parameters (RIFLE-I & F, MAP, IE, APACHE II scores) at RRT initiation, early and late group as compared with intermediate group, indications for RRT (including azotemia, fluid overload & oliguria, and hyperkalemia & acidosis), as well as surgery types (including neurosurgery, chest surgery, CVS, abdominal surgery, and other surgery). Duration for analysis is measured using calendar days from RRT initiation to end point (mortality or discharge).

a hazard for patients in intermediate group  =  1.0;

b every increment of 1 year or point;

c hazard for woman  =  1.0; hazard for patients without DM. ^c^, liver cirrhosis.

d ECMO.

e initial neurological dysfunction.

f sepsis at RRT initiation.

g = 1.0.

h data measured at RRT initiation.

**Abbreviations:** APACHE-II, Acute Physiology and Chronic Health Evaluation II; CI, confidence interval; CKD, chronic kidney disease; DM, diabetes mellitus; ECMO, extracorporeal membrane oxygenation; IE, Inotropic equivalent; MAP, mean arterial pressure; RRT, renal replacement therapy.

When compared with IG, LG [hazard ratio (HR), 1.527; 95% confidence interval (CI), 1.152–2.024; *P = *0.003] independently predicted in-hospital mortality, but EG (HR, 1.213; 95% CI, 0.914–1.612; *P = *0.182) did not. Other independent predictors included age (HR, 1.014; 95% CI, 1.006–1.021), DM (HR, 1.279; 95% CI, 1.022–1.601; *P = *0.031), cirrhosis (HR, 2.147; 95% CI, 1.421–3.242), ECMO support (HR, 1.811; 95% CI, 1.391–2.359), initial neurological dysfunction (HR, 1.448; 95% CI, 1.107–1.894; *P* = 0.007), sepsis at RRT initiation (HR, 1.939; 95% CI, 1.536–2.449), as well as MAP (HR, 0.988; 95% CI, 0.981–0.995), IE (HR, 1.006; 95% CI, 1.001–1.012; *P* = 0.013), and APACHE II scores (HR, 1.055; 95% CI, 1.037–1.073) at RRT initiation. (All *P*<0.001 except otherwise stated).

### Predictors for Entering EG and LG

Basic demographic data, comorbidities, clinical parameters (MAP, IE, APACHE II) at ICU admission, ECMO, MV, initial neurological dysfunction, sepsis at RRT, indications for RRT, and surgery types were put into logistic regression method to determine the predictors for entering EG and LD. Baseline CKD [odds ratio (OR), 1.805; 95% CI, 1.188–2.740; *P* = 0.006], undergoing CVS (OR, 2.816; 95% CI, 1.901–4.173), with higher ICU APACHE II scores (OR, 1.113; 95% CI, 1.076–1.152) along with fluid overload (OR, 3.754; 95% CI, 1.319–10.688; *P* = 0.013) were predictors for entering EG. Whereas those who undergoing chest surgery (OR, 4.236; 95% CI, 2.072–8.659) with higher ICU MAP (OR, 1.009; 95% CI, 1.000–1.019; *P* = 0.050), receiving MV support (OR, 4.497; 95% CI, 1.809–11.180; *P* = 0.001), complicating with sepsis (OR, 1.914; 95% CI, 1.180–3.106; *P* = 0.009) and azotemia (OR, 2.039; 95% CI, 1.356–3.064; *P* = 0.001) had higher probability to receive RRT at a later day (≧4 days). (All *P*<0.001 except otherwise stated).

## Discussion

To our knowledge, this is the first investigation revealing the U-curve association between the timing of RRT initiation after the ICU admission and in-hospital mortality in postoperative AKI.

### Independent Predictors of In-hospital Mortality

Compatible with other studies [30], [31], the in-hospital mortality rate in our population was 58.5%. The current analysis also determined several independent predictors of the in-hospital mortality (table 3). A large body of evidence has shown significant associations between increased mortality rate and older age, DM, liver dysfunction, neurological dysfunction, higher APACHE II scores, and the presence of sepsis [2], [12], [31], [32], [33]. Also a BP that falls below a predetermined threshold is associated with a higher IE and poorer prognosis in the critically ill patients [34].

ECMO, a temporary cardiopulmonary support, may be associated with ischemia/reperfusion (I/R) kidney injury which is a major cause of postoperative AKI in ECMO patients [35]. Due to the interaction of numerous factors, including immune dysregulation, platelet dysfunction, and RRT-related hemodynamic instability and infection, the I/R AKI is associated with a high mortality rate ranging from 40–80% in those with ECMO needing RRT [36], [37].

Additionally, late initiation of RRT could also independently predict in-hospital mortality. In our study, the LG had a higher proportion of chest surgery, which was thought to more likely lead to hospital-acquired infections and the “septic AKI” [38]. The patients in the LG could have either late onset AKI or delayed RRT for AKI that had been present for a longer duration. Late development of AKI is usually related to sepsis, while both the hospital-acquired infection [39] and septic AKI [38] are associated with higher in-hospital mortality. The longer duration of AKI [40] and later RRT initiation [10], [41] are also shown to have a significant adverse impact on the patient survival.

It’s worthy of mention that the existing CKD is recognized as a potent predictor of AKI and may affect the patient outcome [42], [43]. In our study, the baseline CKD was associated with higher proportion of “hyperkalemia or acidosis” (*P* = 0.046) as the indication for RRT initiation. It was also found to be an independent predictor for entering EG, but however, not for the in-hospital mortality.

### Contributors of the Two Peaks in the U-curve

The first peak of the U-curve represented the combined effect of poor clinical conditions (higher APACHE II scores and IE, but lower MAP) and the I/R injuries related to ECMO. Whereas older age, sepsis with subsequent complications, and later initiation of RRT were responsible for the second peak in the curve.

### Comparisons with Previous Works- The Role of Temporal Timing of RRT Initiation

A prospective, multicenter, observational study by Bagshaw *et al*. [9], which included 1238 patients with equal proportions of surgical and medical admissions, evaluated the relationship between the timing of the RRT initiation in severe AKI and the prognoses. The timing of the RRT was defined by different means including various levels of uremic toxins and the period from the ICU admission to the start of RRT. All patients were categorized into the early (<2 days), delayed (2–5 days), and late (>5 days) RRT groups. Similar to our results, the study found that patients in the late RRT group were older and had a higher proportion of patients affected by sepsis. These patients were also prone to be more azotemic and nonoliguric at RRT initiation. However, unlike our investigation in which only approximately 40% patients were in the EG, the majority (64%) of patients in that study underwent RRT within the first day of the ICU admission. Because the interval from hospital admission to the ICU admission was only one day [44], the “2-days interval” between hospitalization and the RRT initiation may be suggestive of a relatively late hospital admission. The study also hinted at the different clinical practice patterns applied among the various areas of medicine and/or surgery and the difficulty of recognizing the exact time point of the renal insult in the medical patients.

Another prospective study that evaluated the association between the timing of RRT initiation and the outcomes was performed by Iyem *et al*. [45]. This study included 185 patients with RRT-requiring postoperative AKI after open-heart surgery. They found no survival difference between early RRT (immediate RRT initiation for AKI, n = 95) and late RRT (RRT start after 48 hours of AKI development, n = 90). However, the study population (all CVS patients) and the initial conditions (no statistical significance between the two groups) in this study were different from our own (only half underwent CVS and higher initial severity of illness in the EG group). CVS is associated with different risks and patient outcomes than those associated with noncardiac surgeries [46]. Additionally, the Iyem *et al*. study only categorized patients into 2 groups, in which the late RRT group in the temporal sense was equivalent to our IG and LG combined. However, mixing the two groups may generate different results.

More recently, a retrospective, multicenter study [47] that included 203 patients with post-CVS AKI demonstrated that early RRT (≦3days after CVS) improved the survival benefit when compared to the survival of the late RRT (>3 days) group. Although the patient population and the categorization of the patients differed from our own, the results were consistent in the two studies if we took the EG and IG together as the equivalent of the early RRT group in former study.

Several significant aspects of our study must be addressed. First, because the main hospital where the study took place is a leading teaching hospital of CVS and ECMO, the proportion of the patients with ECMO support was higher than in other studies. Second, the APACHE II scores in current study were significantly lower than those in other studies which are conducted in the ICU predominantly admitting medical patients according to the traditional ICU admission criteria such as sepsis, shock, or respiratory failure. In our study, all were surgical patients, and nearly half underwent elective surgeries. Many of them admitted into ICU for surgery-related complications monitor but not for the critical illness. Besides, the use of ECMO may underestimate the severity scores because some parameters such as blood pressure under ECMO support were pseudo-normalized. [48] Third, the timing of the RRT initiation should be based on the onset of AKI, which was assumed to coincide with the time point of the surgery. However, this is only true for those undergoing CVS on ECMO support. In the current study, approximately 50% of the patients underwent noncardiac surgeries, resulting in quite a heterogeneous mix of etiologies leading to AKI. Thus, we used the ICU admission date, not the surgery day, as the initial time point. Forth, the study design of categorizing patients by every 2 calendar days (0–1, 2–3, ≧4 days) might not be the best method as it does not take factor in the role of the pathophysiology. Due to the significant factor “septic AKI” plays in the LG, determining a time point to differentiate septic from nonseptic AKI would include the pathophysiology in the analysis. However, the various intervals between the ICU admissions and RRT initiations due to the development of septic AKI (median, 2 days; inter-quartile ranges, 0 to 6 days) [38] make deciphering the optimal time point difficult.

### Limitations

Several limitations of this study should be addressed. First, as an observational study, it is potentially prone to bias. Second, the study was performed with a limited number of surgical patients predominantly undergoing CVS. Thus the results may not serve as a representative sample of the AKI patients without surgery or with different proportions of surgery types throughout the world. Third, since the term “postoperative AKI” not yet has a universal definition, we use its broad meaning to represent the AKI occurring in the surgical patients. However, we should address that the AKI in our patients (especially in LG) may be resulted from complications occurred later during admission rather than the surgery itself. Forth, the study was designed to enroll only patients with postoperative AKI requiring the RRT. Thus, we cannot compare current data with those whose renal function recovered without the RRT or those who died before the RRT initiation. Fifth, one of the predefined indications for RRT initiation, CVP>12 mmHg maybe not an adequate proxy for fluid overload. To address the issue of fluid overload more clearly, we provided the “net fluid balance” at RRT initiation of the three groups. However, the “net fluid balance” didn’t show its significance in the comparison between survivors and non-survivors as well as in the Cox proportional hazards model for outcome analysis.

Furthermore, multicenter randomized clinical trials based on a dependable compound AKI severity scoring system, which contain some early predictive biomarkers such as urine neutrophils gelatinase-associated lipocalin or even genetic risk factors [49], are warranted to confirm our findings.

### Conclusions

This study found the U-curve association between the timing of RRT initiation after the ICU admission and the outcomes in postoperative AKI. The first peak of mortality rate was related to the poor clinical conditions and the I/R injuries related to ECMO support, and the second peak was associated with older age, sepsis, and later initiation of RRT. Current findings remind physicians to be alert to patients with certain factors that may predict poor prognoses after the RRT initiation.

## Supporting Information

Table S1 **Comparisons of demographic data and clinical parameters among the three groups (complete data).**
(DOC)

Table S2 **Comparisons of demographic data and clinical parameters between survivors and non-survivors. (complete data).**
(DOC)
