# Supplementary material for: U-Curve Association between Timing of Renal Replacement Therapy Initiation and In-Hospital Mortality in Postoperative Acute Kidney Injury
Source: PLoS One. 2012 Aug 28;7(8):e42952. doi: 10.1371/journal.pone.0042952 (PMC3429468; doi:10.1371/journal.pone.0042952)
Supplement: Table S1 — Comparisons of demographic data and clinical parameters among the three groups (complete data). (DOC) [file pone.0042952.s001.doc]

**Table S1. Comparisons of demographic data and clinical parameters among the three groups (complete data).**

| **Variable** | **EG ∣**  **(*n* =**256**)** | **IG**  **(*n* =**180**)** | **LG∥**  **(*n* =**212**)** | **P-value** |
| --- | --- | --- | --- | --- |
| **Demographic data** |  |  |  |  |
| Age, years | 61.3 ± 14.7 | 62.2 ± 16.5 | 65.7 ± 16.5* | 0.009 |
| Man | 169 (66.0) | 114 (63.3) | 135 (63.7) | 0.808 |
| DM | 90 (35.2) | 60 (33.3) | 68 (32.1) | 0.777 |
| CKD | 139 (54.3) ** | 78 (43.3) | 90 (42.5) | 0.015 |
| Hypertension | 127 (49.6) | 92 (51.1) | 99 (46.7) | 0.668 |
| Heart failure | 12 (4.7) | 11 (6.1) | 13 (6.1) | 0.738 |
| Cirrhosis | 9 (3.5)** | 19 (10.6) | 13 (6.1) | 0.012 |
| Initial neurological dysfunction | 55 (21.5) | 27 (15.0) | 44 (20.8) | 0.204 |
| Sepsis at RRT | 43 (16.8) | 31 (17.2) | 68 (32.1)** | <0.001 |
| Sepsis at death^￥^ | 81 (53.6) | 52 (61.2) | 110 (77.5)** | <0.001 |
| ECMO support | 92 (35.9)** | 32 (17.8) | 43 (20.3) | <0.001 |
| Mechanical Ventilation | 240 (93.8) | 163 (90.6) | 204 (96.2) | 0.071 |
| CVVH as initial mode | 197 (77.0)** | 100 (55.6) | 113 (53.3) | <0.001 |
| Elective surgery | 98 (38.3) | 76 (42.2) | 94 (44.3) | 0.400 |
| Hospital stay, days | 43.0 ± 43.6* | 52.5 ± 53.7 | 62.8 ± 50.9 | <0.001 |
| ICU to RRT, days | 0.6 ± 0.5** | 2.6 ± 0.7 | 17.9 ± 24.5** | <0.001 |
| RRT to death/discharge, days | 31.2 ± 33.9 | 38.8 ± 43.5 | 34.6 ± 36.5 | 0.111 |
| Surgery to`ICU admission | 1.9 ±16.2 | 3.0 ±10.1 | 1.0 ±14.8 | 0.360 |
| **Surgery category** |  |  |  | <0.001 |
| Neurosurgery | 4 (1.6) | 7 (3.9) | 9 (4.2) | 0.189 |
| Chest surgery | 5 (2.0)* | 12 (6.7) | $1 (19.3)** | <0.001 |
| Cardiovascular surgery | 185 (72.3)** | 86 (47.8) | 76 (35.8)* | <0.001 |
| Abdominal surgepy | 51 (19.9)** | 63 (35.0) | 69 (32.5) | 0.001 |
| Others | 11 (4.3) | 12 (6.7) | 17 (8.0) | 0.237 |
| **Data at ICU admission** |  |  |  |  |
| MAP, mmHg | 79.9 ± 22.1** | 87.8 ± 23.2 | 90.1 ± 21.5* | <0.001 |
| BUN, mg/dL | 46.2 ± 28.3** | 38.1 ± 25.9 | 38.3 ± 29.9 | 0.002 |
| Creatinine, mg/dL | 2.8 ± 1.9* | 2.4 ± 1.6 | 1.8 ± 1.2** | <0.001 |
| eGFR, ml/min/1.73m^2^ | 33.4 ± 20.4** | 41.2 ± 27.0 | 53.5 ± 31.5** | <0.001 |
| Potassium, mmol/L | 4.4 ± 0.9** | 4.1 ± 0.7 | 4.0 ± 0.7 | <0.001 |
| CVP, mmHg | 12.4 ± 5.9** | 10.6 ± 4.9 | 10.5 ± 5.1 | <0.001 |
| IE, mcg/kg/min | 15.1 ± 22.3* | 10.7 ± 18.8 | 5.3 ± 11.2** | <0.001 |
| GCS scores | 11.4 ± 4.9** | 12.9 ± 3.9 | 13.1 ± 3.5 | <0.001 |
| APACHE II scores | 13.1 ± 6.5** | 10.3 ± 6.4 | 9.3 ± 5.4 | <0.001 |
| SOFA scores | 10.0 ± 3.5** | 8.0 ± 3.0 | 6.7 ± 3.5** | <0.001 |
| **Data at RRT initiation** |  |  |  |  |
| RIFLE-I & -F | 69 (27.0)** | 119 (66.1) | 142 (67.0) | <0.001 |
| Net fluid balance, %BW | 2.6 ± 14.0 | 5.8 ± 13.8 | 5.9 ± 14.6 | 0.017 |
| MAP, mmHg | 76.5 ± 16.8** | 84.5 ± 16.1 | 82.0 ± 15.3 | <0.001 |
| WBC, 10^3^/uL | 12.1 ± 6.7** | 14.6 ± 7.8 | 14.3 ± 7.3 | <0.001 |
| Hemoglobin, g/dL | 11.5 ± 5.5 | 10.7 ± 2.9 | 10.1 ± 1.7** | 0.001 |
| BUN, mg/dL | 49.0 ± 28.7** | 58.6 ± 25.9 | 90.1 ± 43.3** | <0.001 |
| Creatinine, mg/dL | 3.0 ± 1.8** | 3.8 ± 1.9 | 3.3 ± 1.7* | <0.001 |
| eGFR, ml/min/1.73m^2^ | 29.7 ± 19.3** | 21.7 ± 15.7 | 26.6 ± 22.8* | <0.001 |
| CVP, mmHg | 13.4 ± 5.6 | 14.5 ± 5.4 | 13.7 ± 5.3 | 0.136 |
| IE, mcg/kg/min | 19.9 ± 22.1* | 14.9 ± 19.7 | 9.6 ± 12.5** | <0.001 |
| GCS scores | 11.3 ± 4.8 | 12.2 ± 4.3 | 11.1 ± 4.4 | 0.169 |
| APACHE II scores | 12.9 ± 6.6 | 12.2 ± 6.2 | 12.8 ± 5.7 | 0.535 |
| SOFA scores | 11.4 ± 3.6 | 11.5 ± 3.6 | 11.3 ± 3.9 | 0.856 |
| **Indications for RRT** |  |  |  |  |
| Azotemia with uremic symptoms ^a^ | 115 (44.9) | 98 (54.4) | 145 (68.4)** | <0.001 |
| Fluid overload ^b^ | 163 (63.7) | 122 (67.8) | 123 (58.0) | 0.131 |
| Oliguria or anuria ^c^ | 237 (92.6) | 162 (90.0) | 144 (67.9)** | <0.001 |
| Hyperkalemia or acidosis ^d^ | 30 (11.7) | 16 (8.9) | 34 (16.1) | 0.089 |
| **In-hospital mortality** | 151 (59.0)* | 85 (47.8) | 14 (67.0)** | 0.001 |
| **RRT wean-off** | 82 (32.0)* | 79 (43.9) | 67 (31.8)* | 0.017 |

**Notes:** EG, ≦ 1 day; IG, 2-3 days; LG, ≧4 days between ICU admission and RRT initiation. Values are presented as mean ± standard deviation or number (percentage) unless otherwise stated. P-value was calculated using Kruskal-Wallis Rank Sum Test , Wilcoxon Rank Sum Tests in two sample comparison with [Bonferroni correction](http://en.wikipedia.org/wiki/Bonferroni_correction) and Fisher's Exact Test for count data. The data at ICU admission and RRT initiation included MAP, WBC, hemoglobin, platelet, BUN, creatinine, eGFR, albumin, sodium, potassium, CVP, IE, GCS, APACHE II, SOFA scores. To save the space, only those with significant differences or important information were listed in the table.

^￥^the percentage was calculated dividing by number of the deaths;

**∣,** EG versus IG; **∥,** LG versus IG; ***,** P<0.05; ****,** P<0.01.

^a^ azotemia was defined as BUN > 80 mg/dl and creatinine > 2 mg/dl; ^b^ fluid overload means CVP>12mmHg; ^c^ oliguria was defined as urine output <100 ml/8hr with diuretics use; ^d^ hyperkalemia denotes serum potassium>5.5 mmol/l, acidosis denotes pH < 7.2 in arterial blood. RRT wean-off, cessation from RRT for at least 30 days

**Abbreviations:** APACHE II, Acute Physiology and Chronic Health Evaluation II; BMI, body mass index; BUN, blood urea nitrogen; CKD, chronic kidney disease; CVP, central venous pressure; CVVH, continuous venous-venous hemofiltration; DM, diabetes mellitus; ECMO, extracorporeal membrane oxygenation; EG, early group; eGFR, estimated glomerular filtration rate; GCS, Glasgow Coma Scale; ICU, intensive care unit; IE, inotropic equivalent; IG, intermediate group; LG, late group; MAP, mean arterial pressure; RRT, renal replacement therapy; SOFA, Sequential Organ Failure Assessment; WBC, white blood cell.
