# Supplementary material for: U-Curve Association between Timing of Renal Replacement Therapy Initiation and In-Hospital Mortality in Postoperative Acute Kidney Injury
Source: PLoS One. 2012 Aug 28;7(8):e42952. doi: 10.1371/journal.pone.0042952 (PMC3429468; doi:10.1371/journal.pone.0042952)
Supplement: Table S2 — Comparisons of demographic data and clinical parameters between survivors and non-survivors. (complete data). (DOC) [file pone.0042952.s002.doc]

**Table S2. Comparisons of demographic data and clinical parameters between survivors and non-survivors. (complete data).**

|  | **Survivors**  (n=269) | **Non-survivors**  (n=379) | **P-value** |
| --- | --- | --- | --- |
| **Demographic data** |  |  |  |
| Age, years | 61.2 ± 15.8 | 64.3 ± 15.8 | 0.014 |
| Man | 180(66.9) | 238 (62.8) | 0.317 |
| DM | 86 (39.4) | 132 (34.8) | 0.500 |
| CKD | 152 (56.5) | 155 (40.9) | <0.001 |
| Hypertension | 140 (52.0) | 178 (56.0) | 0.190 |
| Heart failure | 19 (7.1) | 17 (4.5) | 0.167 |
| Cirrhosis | 14 (5.2) | 27 (7.1) | 0.413 |
| Initial neurological dysfunction | 29 (10.8) | 97 (25.6) | <0.001 |
| Sepsis at RRT | 27 (10.0) | 115 (30.3) | <0.001 |
| ECMO support | 49 (18.2) | 118 (31.1) | <0.001 |
| Mechanical Ventilation | 245 (91.1) | 362 (95.5) | 0.032 |
| CVVH as initial mode | 142 (52.8) | 268 (70.7) | <0.001 |
| Elective surgery | 114 (42.4) | 154 (40.6) | 0.686 |
| Hospital stay, days | 74.2 ± 57.1 | 36.1 ± 35.7 | <0.001 |
| ICU to RRT, days | 5.2 ± 8.9 | 7.8 ± 19.1 | 0.037 |
| RRT to death/discharge, days | 56.9 ± 45.1 | 18.3 ± 19.2 | <0.001 |
| **Surgery category** |  |  | 0.051 |
| Neurosurgery | 13 (4.8) | 7 (1.8) | 0.038 |
| Chest surgery | 16 (5.9) | 42 (11.1) | 0.026 |
| Cardiovascular surgery | 145 (53.9) | 202 (53.3) | 0.936 |
| Abdominal surgery | 79 (29.4) | 104 (27.4) | 0.596 |
| Others | 16 (5.9) | 24 (6.3) | 0.870 |
| **Data at ICU admission** |  |  |  |
| Net fluid balance, %BW | 4.4 ± 11.3 | 4.7 ± 16.0 | 0.765 |
| MAP, mmHg | 88.4 ± 22.2 | 83.5 ± 22.5 | 0.006 |
| Creatinine, mg/dL | 2.7 ± 1.9 | 2.1 ± 1.5 | <0.001 |
| eGFR, ml/min/1.73m^2^ | 37.6 ± 25.3 | 45.4 ± 28.7 | <0.001 |
| Albumin, g/dL | 3.0 ± 0.7 | 2.9 ± 0.7 | 0.050 |
| IE, mcg/kg/min | 8.5 ± 14.2 | 12.2 ± 21.3 | 0.013 |
| GCS scores | 13.4 ± 3.3 | 11.6 ± 4.7 | <0.001 |
| APACHE II scores | 9.8 ± 5.6 | 12.1 ± 6.7 | <0.001 |
| SOFA scores | 7.9 ± 3.2 | 8.7 ± 3.9 | 0.004 |
| **Data at RRT initiation** |  |  |  |
| RIFLE-I & -F | 143 (53.2) | 187 (49.3) | 0.340 |
| Duration between ICU to RRT |  |  | 0.001 |
| ≦ 1 day | 105 (39.0) | 151 (39.8) |  |
| 2-3 d days | 94 (34.9) | 86 (22.7) |  |
| ≧4 days | 70(26.0) | 142 (37.5) |  |
| MAP, mmHg | 85.4 ± 15.7 | 77.0 ± 16.2 | <0.001 |
| Creatinine, mg/dL | 3.8 ± 2.0 | 3.0 ± 1.6 | <0.001 |
| eGFR, ml/min/1.73m^2^ | 22.6 ± 16.3 | 29.2 ± 21.7 | <0.001 |
| IE, mcg/kg/min | 11.3 ± 16.5 | 17.9 ± 20.5 | <0.001 |
| GCS scores | 13.1 ± 3.4 | 10.2 ± 4.9 | <0.001 |
| APACHE II scores | 10.5 ± 5.2 | 14.2 ± 6.4 | <0.001 |
| SOFA scores | 10.0 ± 3.1 | 12.4 ± 3.8 | <0.001 |
| **Indications for RRT** |  |  |  |
| Azotemia with uremic symptoms ^a^ | 153 (56.9) | 205 (54.1) | 0.521 |
| Fluid overload ^b^ | 154 (57.2) | 254 (67.0) | 0.013 |
| Oliguria or anuria ^c^ | 227 (84.4) | 316 (83.4) | 0.676 |
| Hyperkalemia or acidosis ^d^ | 25 (9.3) | 55 (14.6) | 0.052 |
| **RRT wean-off** | 200 (74.3) | 28 (7.4) | <0.001 |

**Notes:** Values are presented as mean ± standard deviation or number (percentage) unless otherwise stated. P-value was calculated using Wilcoxon Rank Sum Tests for continuous data and Fisher's Exact Test for count data. Our data at ICU admission and RRT initiation included MAP, WBC, hemoglobin, platelet, BUN, creatinine, eGFR, albumin, sodium, potassium, CVP, IE, GCS, APACHE II, SOFA scores. To save the space, only those with significant differences or important information were listed in the table.

^a^ azotemia was defined as BUN > 80 mg/dl and creatinine > 2 mg/dl; ^b^ fluid overload means CVP>12mmHg; ^c^ oliguria was defined as urine output <100 ml/8hr with diuretics use; ^d^ hyperkalemia denotes serum potassium>5.5 mmol/l, acidosis denotes pH < 7.2 in arterial blood. RRT wean-off, cessation from RRT for at least 30 days.

**Abbreviations:** APACHE II, Acute Physiology and Chronic Health Evaluation II; BMI, body mass index; BUN, blood urea nitrogen; CKD, chronic kidney disease; CVP, central venous pressure; CVVH, continuous venous-venous hemofiltration; DM, diabetes mellitus; ECMO, extracorporeal membrane oxygenation; EG, early group; eGFR, estimated glomerular filtration rate; GCS, Glasgow Coma Scale; ICU, intensive care unit; IE, inotropic equivalent; IG, intermediate group; LG, late group; MAP, mean arterial pressure; RRT, renal replacement therapy; SOFA, Sequential Organ Failure Assessment; WBC, white blood cell.
